# Insulin-like growth factor-I (IGF-I) and thioredoxin are differentially expressed along the reproductive tract of the ewe during the oestrous cycle and after ovariectomy

**DOI:** 10.1186/1751-0147-48-5

**Published:** 2006-06-12

**Authors:** Elize van Lier, Ana Meikle, Håkan Eriksson, Lena Sahlin

**Affiliations:** 1 Animal and Forage Sciences Department, Faculty of Agriculture, Universidad de la República, Montevideo, Uruguay; 2 Department of Molecular and Cellular Biology, Veterinary Faculty, Universidad de la República, Montevideo, Uruguay; 3 Division for Reproductive Endocrinology, Department of Woman and Child Health, Karolinska Institutet, Stockholm, Sweden

## Abstract

Insulin-like growth factor-I (IGF-I) and thioredoxin are regulated by gonadal steroids in the female reproductive tract of many species. Oestradiol regulates IGF-I and thioredoxin mRNA levels in the reproductive tract of prepubertal lambs. The physiological status (different endocrine environment) may affect the sensitivity of the reproductive tract to oestradiol and progesterone. We studied the effects of different endocrine milieus (late-follicular and luteal phases of the oestrous cycle, and ovariectomy before or after puberty) on the expression of IGF-I, thioredoxin, oestrogen receptor α (ERα) and progesterone receptor (PR) in sheep. The mRNA levels were determined by a solution hybridisation technique. In the uterus the levels of ERα, PR and thioredoxin mRNA were higher in the late-follicular phase group than in the other three groups, and IGF-I mRNA was high during both the late-follicular and the luteal phases. In the cervix only PR mRNA was significantly higher in the ewes in the late-follicular phase than in the other groups. In the oviducts the levels of thioredoxin and ERα mRNA were highest in the ovariectomised adult ewes, and thioredoxin mRNA was higher than the levels found in the ewes in the late-follicular phase. The IGF-I mRNA levels in the oviduct did not differ between any of the groups. The transcripts of IGF-I, thioredoxin, ERα and PR, varied according to the physiological status and also along the female reproductive tract, suggesting that the regulation of the mRNA levels of these factors by the steroid environment is tissue specific.

Koncentrationen av insulin-like growth factor-I (IGF-I) och thioredoxin regleras hos många arter i honors reproduktionsorgan av könssteroider. Sålunda reglerar östradiol IGF-I och thioredoxin mRNA i reproduktionsorganen hos prepubertala lamm. Djurets fysiologiska status (dvs den endokrina miljön) kan påverka känsligheten hos reproduktionsorganen för östradiol och progesteron. Vi studerade effekterna av olika endokrina miljöer (sen follikelfas och lutealfas i östruscykeln, samt ovariektomi före och efter puberteten) på uttrycket av IGF-I, thioredoxin, östrogenreceptor α (ERα) och progesteronreceptorn (PR) hos får. Lösningshybridisering användes för att bestämma mRNA nivåerna. I livmodern var mRNA koncentrationen för ERα, PR och thioredoxin högre i sen follikelfas än i de andra tre grupperna och IGF-I mRNA nivån var hög både under sen follikelfas och i lutealfas. PR mRNA i cervix var signifikant högre hos tackorna under sen follikelfas än i de andra grupperna. I äggledarna var mRNA nivåerna av thioredoxin och ERα högst i de djur som ovariektomerats som vuxna, och thioredoxin mRNA var högre än hos tackorna under sen follikelfas. Det förelåg ingen skillnad vad gäller IGF-I mRNA nivåerna i äggledaren mellan någon av grupperna. IGF-I, thioredoxin, ERα och PR mRNA nivåerna varierade beroende på fysiologisk status och morfologisk lokalisation i reproduktionsorganen. Detta tyder på att steroidhormonernas reglering av dessa faktorers mRNA uttryck också är vävnadsspecifik.

## 1. Introduction

Oestrogens and progestins secreted by the ovaries are the major modulators of the female reproductive tract functions. Their actions are primarily mediated via binding to specific intracellular receptors in the target cells, and subsequent stimulation of gene transcription [1]. The tissue response is determined by both the concentration of the sex steroids in the circulation and the concentration of their high affinity receptors in the tissues [2]. Furthermore, the steroids regulate the sex steroid receptor content along the reproductive tract in a specific manner [3].

Growth factors (*e.g*. insulin-like growth factor-I, IGF-I) and other bioactive molecules, such as thioredoxin, mediate many of the sex steroid actions on the reproductive tract [4-6]. IGF-I has multiple effects on cellular growth and metabolism [7], and is the major paracrine growth factor secreted by uterine stroma [8]. IGF-I promotes cellular mitosis and differentiation in the endometrium [9]. There is direct evidence that IGF-I plays a significant role in reproductive tract development [10]. In the uterus, IGF-I expression is mainly regulated by oestrogens [9,11,12]. Oestradiol treatment induced IGF-I mRNA expression in the oviducts, uterus and cervix of prepubertal ewes [6], while progesterone treatment affected IGF-I mRNA expression only in the uterus [13].

Thioredoxin is a small multifunctional protein (12 kDa) that acts as a hydrogen donor to the enzyme ribonucleotide reductase that reduces ribonucleotides to deoxyribonucleotides and, thus, is essential for DNA synthesis [14]. The biological functions of thioredoxin in the uterus are likely to be the same as in other cells, and are probably coupled to the mitogenic activity in the uterus and the subsequent DNA, RNA and protein synthesis [15,16]. Oestradiol increases the thioredoxin mRNA levels in the rat uterus, and the oestradiol level is positively correlated to cervical thioredoxin mRNA levels in non-pregnant women [17,18]. Studies in sheep are scarce, but oestradiol has been shown to stimulate thioredoxin expression also in the oviducts, uterus and cervix of prepubertal lambs [6].

The regulation of IGF-I and thioredoxin expression along the reproductive tract of intact prepubertal lambs has been studied after oestradiol/progesterone treatment [6,13]. Most studies on oestradiol and progesterone regulation of uterine gene expression have been conducted using ovariectomised ewes or lambs, or anoestrous ewes, with steroid replacement/treatment. These experimental paradigms do not give a clear picture about gene expression under naturally occurring conditions. The endocrine environment differs according to the physiological status of the animal and this may affect the sensitivity of the reproductive tract to oestradiol and progesterone (*e.g*. oestrogen and progesterone receptors) and thus, the biological response of the tissue to these steroid hormones. Since IGF-I and thioredoxin expression is affected by steroid hormones, we hypothesise that the relative levels of the IGF-I and thioredoxin transcripts will change under differing physiological or endocrine conditions along the reproductive tract (oviducts, uterus and cervix). Therefore, our main objective was to determine the expression of IGF-I and thioredoxin in the reproductive tract under different endocrine milieus (late-follicular and luteal phases of the oestrous cycle and ovariectomy before and after puberty), which to our knowledge is the first report in sheep. Oestrogen receptor (ER) and progesterone receptor (PR) expression along the reproductive tract was also determined and was used as an index of tissue sensitivity to steroid hormones [2].

## 2. Materials and methods

### 2.1. Animals and treatments

This experiment was carried out in Uruguay in the breeding season (May). Eighteen intact and ovariectomised (OVX) Corriedale ewes were used. Oestrus was synchronised in the intact ewes (intra-vaginal sponges impregnated with medroxy-progesterone acetate for 12 days). Oestrus was checked twice daily with a ram from 24 until 72 h after sponge withdrawal. Four different groups were formed according to gonadal status and age at ovariectomy: intact ewes in late-follicular phase (OVF, n = 4) and in luteal phase (OVL, n = 4), ovariectomised adult ewes (OVXa, n = 5) and ovariectomised lambs (OVXy, n = 5). The late-follicular phase group was selected to study the effects of endogenous oestradiol on the expression of IGF-I and thioredoxin in the reproductive tract, while the luteal phase group was selected to study the effects of endogenous progesterone. Follicular and luteal phases were selected to investigate the opposite endogenous endocrine profiles (high oestradiol-low progesterone vs. low oestradiol-high progesterone). The ovariectomised adult ewes were included as sex steroids-free control animals in contrast to the natural occurring sex steroid levels during the follicular and luteal phases of the oestrous cycle. The ovariectomized lambs had not gone through puberty and therefore represent pre-exposure expression of the mRNA's investigated. The adult ewes were more than 3 years old, and the lambs were all 8.5 months old, when sacrificed. Ovariectomy was done 5.5 months (November) prior to sacrifice except in three lambs in which it was done two months (March) prior to sacrifice. All of the animals were accustomed to frequent handling. All animal experimentation was performed in compliance with regulations set by the National Board for Laboratory Animals (Swedish University of Agricultural Sciences, Faculty of Veterinary Medicine, Uppsala, Sweden).

The animals were weighed and sacrificed and the reproductive tracts were obtained. Animals of the same group were sacrificed on the same day. Blood samples were collected prior to slaughter to record the hormonal status of the animals. The hormones were analysed by previously validated radioimmunoassays (Coat-A-Count radioimmunoassay kits, Diagnostic Products Corporation, Los Angeles, CA, USA) (progesterone: [19]; oestradiol: [20]). Progesterone was analysed in the samples before sacrifice of each animal, and in the daily samples from the intact ewes from the day of sponge withdrawal onward. All of the samples were run in the same assay, and the intra-assay coefficients of variation for three control samples (low 3.1 nmol/L, medium 28.1 nmol/L and high 45.0 nmol/L) were 0.5%, 8.4% and 0.7%, respectively. The analytical detection limit of the assay was 0.35 nmol/L. Oestradiol was determined in the samples before sacrifice of each animal and in the daily samples from the intact ewes from sponge withdrawal onward. The intra-assay coefficients of variation for three control samples (low 7.1 pmol/L, medium 47.5 pmol/L and high 128.8 pmol/L) were 11.9%, 7.3% and 7.2%, respectively. The corresponding inter-assay coefficients of variation were 15.1%, 12.6% and 10.9%, respectively. The analytical detection limit of the assay was 2.7 pmol/L. Ewes of the OVF group were sacrificed 72 h after sponge withdrawal (*i.e*. 24 h after first observation of oestrus) and ewes of OVL group were sacrificed 12 days after sponge withdrawal. The reproductive tract was dissected and oviducts, uterus and cervix were separated and weighed. One oviduct of each animal was used for analysis and from each cervix approximately 2 to 3 g of tissue was taken from the centre. Since we have previously demonstrated that ER and PR concentrations are different according to the region of the uterine horn studied [20], the uteri were divided in Upper Zone (cranial portion of the uterine cornua beginning at the uterotubal junction); Lower Zone (caudal portion of the uterine cornua adjacent to the uterocervical junction); and Middle Zone (medial to Upper and Lower zones in the uterine cornua). The uterine samples were not further dissected. The tissues were frozen in liquid nitrogen and stored at -80°C until preparation for solution hybridisation analyses.

### 2.2. TNA preparation and mRNA determination

The tissue samples were homogenised and total nucleic acids (TNA) prepared by digestion of the homogenate with proteinase K in a SDS-containing buffer, followed by subsequent extraction as previously described [21]. The DNA content of the TNA samples was determined by a fluorometric assay at the wavelength 458 nm with Hoechst Dye 33258 [22]. A solution hybridisation assay for specific mRNA determinations was used and performed as before [21]. In short: TNA samples were hybridised with *in vitro *^35^S-UTP labelled RNA probes (~20.000 cpm/incubation) at 70°C. Incubations were performed in duplicates. After overnight incubation each sample was treated with an RNase containing buffer, to digest non-hybridised RNA. The following modification was done in order to quantify the mRNAs. The labelled hybrids protected from RNase digestion were precipitated by addition of trichloroacetic acid and collected on filters (Whatman GF/C, Whatman International Ltd, Maidstone, England). The radioactivity on the filters was monitored in a scintillation counter and the results were compared with a standard curve of known amounts of in vitro synthesized mRNA complementary to the probe used. Every set of tissue samples was run in one assay. Results are expressed as amol (10^-18^) mRNA/μg DNA in the TNA samples. The validation of quantification by solution hybridization has been shown previously [23].

### 2.3. Hybridisation probes

The probe used for IGF-I mRNA determinations was derived from a 775 bp RsaI-EcoRI fragment cDNA of the human insulin-like growth factor-I (IGF-I). The fragment was cloned into the HincII and EcoRI sites of a Bluescript KS vector. Restriction of this vector with XhoI allows the synthesis of a cRNA probe. The probe used for the thioredoxin mRNA determinations was derived from a genomic clone of human thioredoxin cDNA [24]. A fragment of 315 bp representing the 105 amino acids in the open-reading frame of the human thioredoxin gene was sub-cloned into a pGEM 3Z vector. Two subclones with opposite orientations were selected and digested with SmaI. Sense and anti-sense RNA were obtained using T7 RNA polymerase. A northern blot where the human derived thioredoxin anti-sense probe was hybridised to ovine RNA exhibited a single band at approximately 600 base-pairs [6]. The probes used for oERα and oPR mRNA determinations were derived from plasmids containing 360 or 314 bp cDNAs from the ovine ER and PR, respectively, and were kindly supplied by Dr. N. Ing, Texas University, TX, USA [25]. Restriction of the vector (pGEM4Z) containing a fragment of the oER cDNA with *Eco*RI allows the synthesis of an anti-sense RNA probe using T7 RNA polymerase. Restriction of the vector (pCRII) containing a fragment of the oPR cDNA with *Hin*dIII allows the synthesis of an anti-sense RNA probe using T7 RNA polymerase.

### 2.4. Statistics

Analysis of variance of the data was done with a statistical package [26] using the MIXED procedure with the Tukey-Kramer test. The two main effects studied were *group *and *tissue*, and their interaction group*tissue, and *animal *was in the error term. The groups within each tissue were compared using orthogonal contrasts. The variables analysed were ERα, PR, IGF-I and thioredoxin mRNA, and the tissue weight of the uteri, oviducts and cervix. No differences were found in the transcripts among the uterine zones (P > 0.10), therefore the data was pooled and presented as whole uterus samples. The level of significance was P < 0.05 and the results are expressed as mean ± SEM.

## 3. Results

### 3.1. Body weight and sex steroid levels at sacrifice

**Figure 1:**
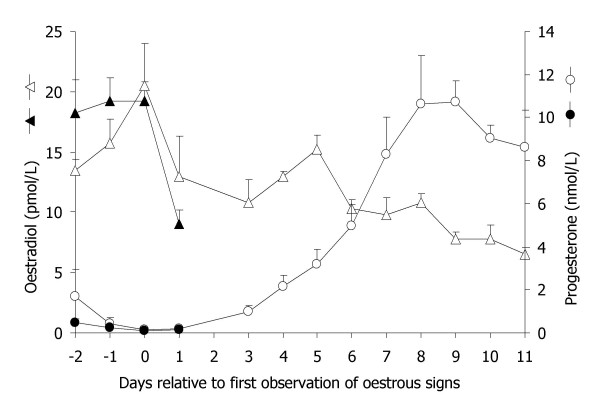
Mean (± SEM) progesterone (nmol/L) and oestradiol (pmol/L) profiles in the intact synchronised ewes before slaughter. Solid triangles and dots: OVF group (slaughtered on day 1 relative to standing oestrus); open triangles and dots: OFL group (slaughtered on day 11 relative to standing oestrus).

Table 1 displays the mean (± SEM) body weight of the animals of each group, as well as the oestradiol and progesterone levels at the time of sacrifice. The lambs weighed significantly less than the adult ewes and the hormones were in accordance with the reproductive status of the animals (Fig. 1, Table 1). All of the intact ewes had shown oestrus signs except for one ewe in the OVL group, nevertheless all of OVL ewes had a corpus luteum at the time of sacrifice. The ovaries of three OVF ewes presented a mature follicle and one OVF ewe had ovulated at the time of sacrifice.

**Table 1:** Animal groups, number (n) of animals per group, mean (± SEM) body weight (BW) (Kg) and the levels of oestradiol (pmol/l) and progesterone (nmol/l) at the time of sacrifice

**Group**	**n**	**BW (kg)**	(SEM)	**Oestradiol (pmol/l)**	(SEM)	**Progesterone (nmol/l)**	(SEM)
OVF	4	39	(2.0)	9.0	(1.2)	0.4	(0.0)
OVL	4	40	(3.0)	6.5	(0.7)	8.6	(1.7)
OVXa	5	42	(1.2)	4.6	(0.7)	0.4	(0.0)
OVXy	5	24	(2.4)	4.5	(0.7)	0.4	(0.0)

OVF: ewes in late-follicular phase, OVL: ewes in luteal phase, OVXa: ewes ovariectomised in adulthood, and OVXy: ovariectomised lambs.

### 3.2. Tissue weights

The uterine weight was higher in the intact ewes than in the ovariectomised animals (P < 0.0001). The weights of cervix and oviducts were highest in the ewes in the late-follicular phase, intermediate in the ewes in the luteal phase and the ovariectomised ewes, and lowest in the ovariectomised lambs (P < 0.01) (Table 2).

**Table 2:** Mean (± SEM) tissue weights (g) in ewes in the late-follicular and luteal phases and ovariectomised ewes and lambs

**Group**	**Oviduct weight**	(SEM)	**Uterine weight**	(SEM)	**Cervical weight**	(SEM)
OVF	3.0^a^	(0.31)	48^a^	(7.5)	17.6^a^	(1.62)
OVL	1.5^b^	(0.18)	37^a^	(3.3)	7.2^b^	(0.36)
OVXa	1.5^b^	(0.12)	12^b^	(1.4)	6.9^b^	(1.04)
OVXy	0.6^c^	(0.10)	3^b^	(0.3)	2.0^c^	(0.30)

The different letters a, b and c in columns indicates statistical significance (P < 0.01). OVF: ewes in late-follicular phase, OVL: ewes in luteal phase, OVXa: ewes ovariectomised in adulthood, and OVXy: ovariectomised lambs.

### 3.3. General effects of treatments on mRNA

The effects of group and tissue were significant as well as their interaction for ERα, PR and IGR-I mRNA (P < 0.0001). For thioredoxin mRNA the effects were also significant (*group*: P = 0.0482; *tissue*: P = 0.0002; and group*tissue: P = 0.0028).

### 3.4. ERα mRNA

**Figure 2:**
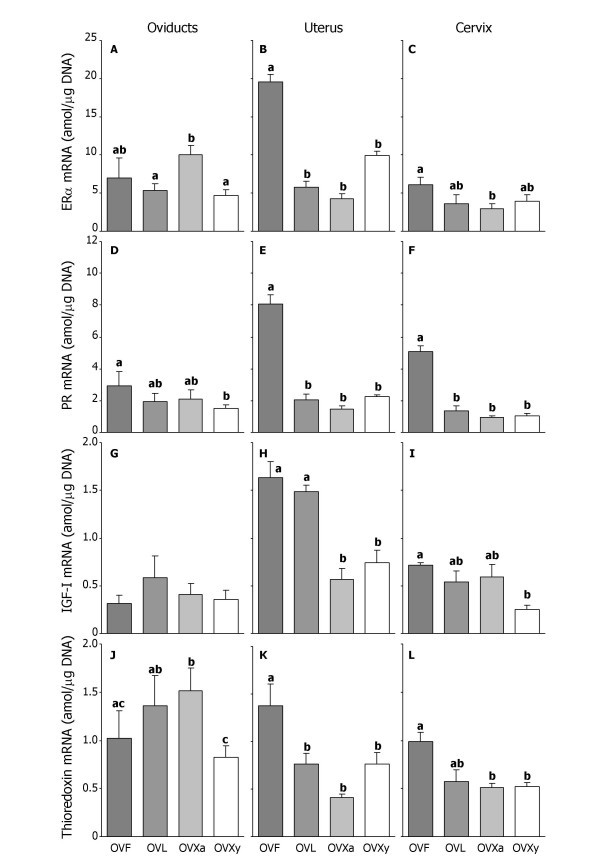
Mean (± SEM) concentrations of ERα (A, B and C), PR (D, E and F), IGF-I (G, H and I), and thioredoxin (J, K and L) mRNA (amol/μg DNA) in oviducts (A, D, G and J), uterus (B, E, H and K) and cervix (C, F, I and L) of ewes in the late-follicular (OVF) and in the luteal (OVL) phase, ovariectomised ewes (OVXa) and ovariectomised lambs (OVXy). Within each graph bars with different letter designations are significantly different, P < 0.05.

In the oviduct, the ovariectomised adult ewes showed the highest levels of ERα mRNA, which were significantly different from those of the ewes in the luteal phase and the ovariectomised lambs (Fig. 2A). In the uterus, however, the levels of ERα mRNA were higher in the late-follicular phase group than in the other three groups (Fig. 2B). In the cervix the ewes in the late-follicular phase had significantly higher levels of ERα mRNA than the ovariectomised ewes (Fig. 2C).

### 3.5. PR mRNA

In the oviducts the PR mRNA levels of the ewes in the late-follicular phase were only significantly higher than those of the ovariectomised lambs (Fig. 2D). In the uterus (Fig. 2E) as well as the cervix (Fig. 2F) the levels of PR mRNA were higher in the late-follicular phase group compared to the other three groups.

### 3.6. IGF-I mRNA

The IGF-I mRNA levels in the oviduct were not different between any of the groups (Fig. 2G). In the uterus the levels of IGF-I mRNA in the intact ewes (OVF and OVL) were higher than those of the ovariectomised ewes and lambs (Fig. 2H). The IGF-I mRNA level in the cervix of the ovariectomised lambs was lower than that of the adult ewes (late-follicular and luteal phase, and ovariectomised) (Fig. 2I).

### 3.7. Thioredoxin mRNA

In the oviducts the ovariectomised adult ewes had the highest levels of thioredoxin mRNA, which were significantly different from the ewes in the late-follicular phase and the ovariectomised lambs (Fig. 2J). In the uterus (Fig. 2K) as well as the cervix (Fig. 2L) the levels of thioredoxin mRNA were higher in the late-follicular phase group than in the other three groups, although the levels of thioredoxin mRNA in the cervix the ewes in the luteal phase were not different from the ones in the late-follicular phase.

## 4. Discussion

This is the first report on IGF-I and thioredoxin expression, studied together with transcripts of the main regulators of uterine function (ERα and PR), under different endocrine environments in the reproductive tract of sheep. The virtue of this study is that no exogenous hormones were used, and therefore, the differences observed reflect the physiological status of the animals. The regulation of the transcripts of IGF-I, thioredoxin, ERα and PR, was most likely dependent on the endogenous endocrine status of the ewes, and for each transcript this regulation varied in the different regions of the reproductive tract.

The oviducts weighed more in the late-follicular phase (Table 2), but this was not associated with the targeted mRNAs since they were either similar (ERα, PR and IGF-I) or lower (thioredoxin) than those of the ovariectomised adult ewes and not different from the ewes in the luteal phase in any of the cases (Figure 2). Similarly, treatment of prepubertal lambs with oestradiol also resulted in a weight increase of the oviducts [20], which was not accompanied with increases of ERα, PR and IGF-I mRNA [13]. If oestrogens were responsible for the higher oviduct weight, the effect on the transcripts was probably short-lived and therefore not seen in this study. This is also supported by another study [6] where IGF-I mRNA and thioredoxin mRNA were maximal 12 h after oestradiol treatment, but no differences were detected after 24 h. Highest IGF-I expression (by *in situ *hybridisation) in ovine oviducts has been observed 48–60 h after a prostaglandin analogue injection coinciding with standing oestrus [27].

The uterine weight of the ewes in the late-follicular phase and the high IGF-I mRNA levels in the uterus confirmed the earlier observations on the effects of oestradiol on uterine weight and IGF-I mRNA in ewe lambs [6,20]. Stevenson *et al*. [4] have found highest IGF-I expression in sheep uterus during oestrus using *in situ *hybridisation, as compared to other phases of the oestrous cycle. IGF-I expression and subsequent uterine growth are induced by oestradiol [12], which was reflected in our study in the high uterine IGF-I mRNA levels of the ewes in the late-follicular phase. No classical oestrogen responsive element (ERE) has been found in the promoter of the IGF-I gene [28], but still oestrogen regulates its expression [6]. In agreement with the role of thioredoxin as a growth promoter of the uterus, its mRNA levels were highest in the ewes in the late-follicular phase. Thioredoxin expression was increased during oestrus also in mouse reproductive tissues [29]. The mRNA levels of ERα and PR in the uterus were as expected, higher in the late-follicular phase than in the luteal phase [30].

In contrast to the uterus, the levels of IGF-I mRNA were not associated with the higher weight of the cervix in the late-follicular phase compared to the other groups (Figure 2). The oestradiol levels had peaked during the two days before tissue collection (Figure 1), and this might explain the lack of differences in cervical IGF-I mRNA levels between the ewes in the late-follicular and luteal phase and the ovariectomised ewes. In an earlier study Sahlin *et al*. [6] reported a prominent though transient induction (only the first 24 h of a three-day-treatment) of IGF-I mRNA in the cervix of oestradiol treated prepubertal lambs. In the present study, ewes in the late-follicular phase (associated with high oestrogen levels) had high steady state levels of ERα, PR and thioredoxin mRNAs in the cervix.

In the ewes in the luteal phase (under influence of progesterone) growth of the uterus was induced, but not of the cervix or the oviducts. Like oestradiol, progesterone induces IGF-I expression and uterine growth [12], and indeed high IGF-I mRNA levels were found in the uterus of ewes in the luteal phase. Increased endometrial expression of IGF-I mRNA has also been observed on days 6 and 8 of pregnancy in sheep, when oestrogens are low and progesterone is high [31]. However, the uterine mRNA levels of ERα, PR and thioredoxin of the ewes in the luteal phase were not different from those of the ovariectomised ewes and lambs. The cervical and oviduct weights of the ewes in luteal phase were similar to that of the ovariectomised ewes and lower than that of the ewes in the late-follicular phase, suggesting that progesterone did not induce proliferation of these tissues. The IGF-I mRNA levels in the cervix and oviducts of the ewes in the luteal phase were not different from those in the ovariectomised ewes. Meikle *et al*. [20] observed a similar differential effect of progesterone on the reproductive tract in immature ewes. In that study uterine weight increased after progesterone treatment while the cervical and oviduct weights remained unchanged, and this was consistent with the increase in IGF-I mRNA only in the uterus [13]. However, Stevenson *et al*. [4] did not observe this increased uterine IGF-I expression during high progesterone levels in the ewe.

The removal of the ovarian source of sex steroids by ovariectomy resulted in a reduction of the cervical, uterine and oviduct weights as compared with the ewes in the late-follicular phase. The cervical weight of the ovariectomised lambs was lower than ovariectomised ewes, but the lambs had not reached adult body weight at ovariectomy, so their reproductive tract was probably not completely developed. Age at ovariectomy (young vs. adult) affected the transcripts: the relative levels of ERα and thioredoxin mRNAs were lower in oviducts, and IGF-I mRNA in the cervix of young animals.

Our results show that the timing and relative levels of the expression of each transcript under different endocrine milieus depend on the organ of the reproductive tract, with the uterus showing the most dramatic and profound changes. Progesterone receptor, IGF-I, and thioredoxin are all markers of oestrogen action in the uterus. Under equal exposure of circulating steroids during the late-follicular phase (*e.g*. oestrogen dominance), the transcripts were stimulated most in the uterus. The regulation of the transcripts was not only tissue specific but also transcript specific. While uterine and cervical PR mRNA seemed to follow the pattern of ERα mRNA very well, the regulation of IGF-I and thioredoxin mRNAs seemed to be less tightly associated to this pattern, suggesting the participation of other factors in their regulation. The differential expression of IGF-I and thioredoxin found in this study could be due to different intracellular machinery within the different cell types, allowing for the specific functions of each organ despite being exposed to the same peripheral hormone levels. Although not addressed in this study, it is known that IGF-I, ERα and PR are expressed in a cell type specific manner and this should be taken into account since hormone sensitivity of the target tissue depends on the specific receptor expression and the cross-talk among different cell types.

In summary, our findings show that IGF-I and thioredoxin expression is regulated in a tissue specific manner along the reproductive tract, as well as ERα and PR expression. The differential transcript expression found in the reproductive tract of sheep under different endocrine environments in which no exogenous hormones were used, suggests that the different responses reported in the literature in hormone treated OVX animals may depend on the initial expression of the transcripts.
